# Quantitative susceptibility mapping in the thalamus and basal ganglia of systemic lupus erythematosus patients with neuropsychiatric complaints

**DOI:** 10.1016/j.nicl.2021.102637

**Published:** 2021-03-22

**Authors:** Marjolein Bulk, Thijs van Harten, Boyd Kenkhuis, Francesca Inglese, Ingrid Hegeman, Sjoerd van Duinen, Ece Ercan, César Magro-Checa, Jelle Goeman, Christian Mawrin, Mark van Buchem, Gerda Steup-Beekman, Tom Huizinga, Louise van der Weerd, Itamar Ronen

**Affiliations:** a Department of Radiology, Leiden University Medical Center, Leiden, The Netherlands; b Department of Human Genetics, Leiden University Medical Center, Leiden, The Netherlands; c Department of Pathology, Leiden University Medical Center, Leiden, The Netherlands; d Department of Rheumatology, Leiden University Medical Center, Leiden, The Netherlands; e Department of Rheumatology, Zuyderland Medical Center, Heerlen, The Netherlands; f Department of Medical Statistics, Leiden University Medical Center, Leiden, The Netherlands; g Department of Neuropathology, Otto-von-Guericke University, Magdeburg, Germany; h Center for Behavioral Brain Sciences (CBBS), Magdeburg, Germany

**Keywords:** Quantitative susceptibility mapping, Neuropsychiatric systemic lupus erythematosus, Neuroinflammation, Basal ganglia, Iron accumulation

## Abstract

Systemic lupus erythematosus (SLE) is an auto-immune disease characterized by multi-organ involvement. Although uncommon, central nervous system involvement in SLE, termed neuropsychiatric SLE (NPSLE), is not an exception. Current knowledge on underlying pathogenic mechanisms is incomplete, however, neuroinflammation is thought to play a critical role. Evidence from neurodegenerative diseases and multiple sclerosis suggests that neuroinflammation is correlated with brain iron accumulation, making quantitative susceptibility mapping (QSM) a potential hallmark for neuroinflammation in vivo. This study assessed susceptibility values of the thalamus and basal ganglia in (NP)SLE patients and further investigated the in vivo findings with histological analyses of postmortem brain tissue derived from SLE patients. We used a 3T MRI scanner to acquire single-echo T_2_*-weighted images of 44 SLE patients and 20 age-matched healthy controls. Of the 44 patients with SLE, all had neuropsychiatric complaints, of which 29 were classified as non-NPSLE and 15 as NPSLE (seven as inflammatory NPSLE and eight as ischemic NPSLE). Mean susceptibility values of the thalamus, caudate nucleus, putamen, and globus pallidus were calculated. Formalin-fixed paraffin-embedded post-mortem brain tissue including the putamen and globus pallidus of three additional SLE patients was obtained and stained for iron, microglia and astrocytes. Susceptibility values of SLE patients and age-matched controls showed that iron levels in the thalamus and basal ganglia were not changed due to the disease. No subgroup of SLE showed higher susceptibility values. No correlation was found with disease activity or damage due to SLE. Histological examination of the post-mortem brain showed no increased iron accumulation. Our results suggest that neuroinflammation in NPSLE does not necessarily go hand in hand with iron accumulation, and that the inflammatory pathomechanism in SLE may differ from the one observed in neurodegenerative diseases and in multiple sclerosis.

## Highlights

• QSM was hypothesized to reflect neuroinflammation in neuropsychiatric SLE.
• Susceptibility values of the basal ganglia are not changed in SLE and NPSLE.
• Neither disease activity nor damage due to SLE correlated with susceptibility values.
• Postmortem brain tissue of SLE patients showed no increase of iron compared to controls.

## 1 Introduction

Systemic lupus erythematosus (SLE) is a female-predominant auto-immune disease characterized by acute or chronic inflammation of multiple organs (Jeltsch-David and Muller, 2014). SLE is the prototype of a systemic disease that can also present with a variety of neurological and psychiatric complaints. The prevalence of major central nervous system involvement in SLE, termed neuropsychiatric (NP) SLE (NPSLE), is estimated to be 12% (Ainiala et al., 2001, Schwartz et al., 2019). Central nervous system involvement leads to a series of uncommon, heterogeneous but potentially severe neurological and psychiatric clinical manifestations.

The precise etiology of NSPLE remains unknown. Currently, the two main underlying pathogenic mechanisms in the brain resulting in NPSLE are thought to be ischemia and inflammation (Hanly, 2014, Sibbitt et al., 2010, Zirkzee et al., 2012). Ischemia is mainly acute in onset and due to the interruption of blood flow of the large and small intracranial vessels in specific brain regions leading to for example cerebrovascular disease. On the other hand, symptoms associated with an underlying autoimmune/inflammatory mechanism are often diffuse cerebral events (e.g. acute confusional state, severe cognitive dysfunction or psychosis) thought to be consequence of neuroinflammation (Hanly, 2014, Sibbitt et al., 2010, Zirkzee et al., 2012, Magro-Checa et al., 2016). Neuroinflammation in NPSLE is thought to be mediated by blood-brain-barrier damage, which facilitates infiltration of leukocytes, autoantibodies and inflammatory mediators into the brain parenchyma, subsequently resulting in an aberrant activation of microglia. However, other interfaces such as the choroid plexus, the site of the blood-cerebrospinal fluid barrier, could also serve as a site of leukocyte and pathogenic antibody transfer into the brain (Schwartz et al., 2019, Hanly, 2014, Sibbitt et al., 2010, Zirkzee et al., 2012). Although there is in-vivo and post-mortem evidence of neuroinflammation in NPSLE (Jeltsch-David and Muller, 2014, Cohen et al., 2017, Curiel et al., 2011, Emmer et al., 2006, Lee et al., 2012), no direct neuroimaging technique has so far contributed to explain the exact mechanism that causes neuroinflammation in NPSLE.

Although it is clear that the central nervous system may be involved in SLE, morphological changes and brain lesions visualized using conventional magnetic resonance imaging (MRI) provide neither a robust link with the clinical symptoms and disease outcome, nor with the pathological mechanisms underlying NPSLE. The so-called clinico-radiological paradox in NPSLE is defined by the presence of brain lesions on MRI in the absence of NP symptoms (Luyendijk et al., 2011). Quantitative MRI research in (NP)SLE is on the rise, but significant works are scarce in the field. Several methods have been used to identify more subtle and diffuse brain changes that may scale with clinical measures related to SLE and NPSLE. Techniques such as magnetization transfer imaging (MTI) and diffusion tensor imaging (DTI) have proven to be useful in identifying microstructural alterations, namely increase in mean water diffusivity/decrease in fractional anisotropy in DTI and decrease in magnetization transfer ratio (MTR), which can be attributed to the effects of neuroinflammation in NPSLE (Ercan et al., 2015, Ercan et al., 2016, Magro-Checa et al., 2016, Corrêa et al., 2018, Kozora et al., 2018, Nystedt et al., 2018, Preziosa et al., 2020). Both techniques, however, only provide indirect evidence for inflammation and possibly also reflect other underlying pathophysiological processes that are not necessarily related to inflammation.

Recently, increasing evidence, particularly from neurodegenerative diseases, suggests that chronic neuroinflammation, characterized by microglia activation and secretion of proinflammatory cytokines, is highly correlated with brain iron accumulation (Bulk et al., 2018, Haider et al., 2014, Simmons et al., 2007, Kaunzner et al., 2019). Thus, quantitative susceptibility mapping (QSM) and other MRI techniques sensitive to brain iron concentrations, could be potential hallmarks for neuroinflammation in vivo. This has already been demonstrated in a wide range of neurological diseases, including cortical grey matter in Alzheimer’s disease (Ayton et al., 2017, van Bergen et al., 2016a), substantia nigra in Parkinson’s disease (Acosta-Cabronero et al., 2017, Langkammer et al., 2016) and striatum in Huntington’s disease (Kaunzner et al., 2019, Langkammer et al., 2013, Macerollo et al., 2014, van Bergen et al., 2016a). Iron accumulation was associated with disease progression in each of these diseases (Ayton et al., 2017, van Bergen et al., 2016b). In addition to neurodegenerative diseases, QSM has also been applied in (neuro)inflammatory diseases such as multiple sclerosis (Haider et al., 2014) and systemic lupus erythematosus (Ogasawara et al., 2016).

In this study, we used QSM to investigate the potential role of iron accumulation in the brain of NPSLE patients and in a group of sex/age matched healthy controls. We assessed susceptibility values of the thalamus and basal ganglia in all subjects and compare the susceptibility values of the healthy controls to those of the patient group as a whole, as well as to subgroups of patients, stratified according to the origin of their neuropsychiatric complaints (related or unrelated to SLE). In addition, we investigated the correlation between susceptibility values of the thalamus and basal ganglia and clinical variables such as SLE disease activity, SLE damage and the presence of autoantibodies. Finally, we further explain our in vivo findings with histological analyses of post-mortem brain tissue of three SLE patients stained for iron and activated glia.

## 2 Methods

### 2.1 Study subjects

All patients were recruited from the Rheumatology department of the Leiden University Medical Center which is the national referral center for NPSLE in the Netherlands SLE patients are referred to the clinic when they have neuropsychiatric complaints. This special role of the department leads to a specific situation whereby the patient population and thus the MRI data we had access to is from SLE patients with NP complaints, some attributable to SLE (NPSLE) or to other factors (non-NPSLE). SLE patients suspected of having NPSLE underwent a standardized multidisciplinary medical examination, as well as extensive neuropsychologic testing, serologic assessment, and brain MRI (Zirkzee et al., 2012). All SLE patients were diagnosed according to the 1982 revised American College of Rheumatology criteria (Hochberg, 1997, Tan et al., 1982). SLE disease activity, a measure for global disease activity, was calculated according the systemic lupus erythematosus disease activity index 2000 (SLEDAI-2K) (Gladman et al., 2002). Permanent and irreversible damage due to SLE was assessed with the systemic lupus erythematosus international collaborating clinics (SLICC)/American College of Rheumatology damage index (SDI) (Gladman et al., 1997). Diagnosis of NPSLE was made by multidisciplinary consensus and, as described by Zirkzee et al. (2012), patients were classified as: (1) NPSLE, or (2) non-NPSLE defined as SLE patients with NP complaints unrelated to SLE as the symptoms were explained by another diagnosis.. During the consensus meeting, different phenotypes were assigned depending on the suspected underlying pathogenetic mechanisms: inflammatory NPSLE or ischemic NPSLE as previously described by our group (Zirkzee et al., 2012, Magro-Checa et al., 2016, Magro-Checa et al., 2016, Luyendijk et al., 2011, Ercan et al., 2016). Furthermore, NPSLE syndromes were assigned according to the 1999 American College Rheumatology NPSLE case definitions (The American College of Rheumatology Nomenclature).

In total, 44 patients with SLE (three males, 41 females, age: 45 ± 13 years) and 20 age-matched healthy volunteers (5 males, 15 females, age: 40 ± 10 years) were included in the study. Of 44 patients with SLE, all had neuropsychiatric complaints, but 29 were classified as non-NPSLE and 15 as NPSLE (seven as inflammatory NPSLE and eight as ischemic NPSLE). Importantly, diagnosis in this study is based on the first evaluation only, and can therefore change during follow-up, based on reassessment including the response to treatment. The demographics of the study and the clinical characteristics of the study population are shown in Table 1. Detailed information on neuropsychiatric symptoms and medication at time of MRI assessment is shown in Supplementary Tables 1 and 2. The study adhered to the Declaration of Helsinki and was approved by the institutional review board of our institution. Written informed consent was obtained from all subjects prior to the study.

**Table 1:** Demographic information of the study population.

	Patients with SLE n = 44	Controls n = 20
Age, mean ± SD years	45 ± 13	40 ± 10
Sex (% female)*	41/44 (93%)	15/20 (75%)
SLEDAI-2 K, median (range)	4 (0–16)	N.A.
SDI, median (range)	1 (0–4)	N.A.
NPSLE (%)	15/44 (34%)	N.A.
Antinuclear antibody	27/32 (84%)	N.A.
Anti-ENA	19/32 (59%)	N.A.
Anti-DNA	13/32 (40%)	N.A.
Anti-RNP U1	8/32 (25%)	N.A.
Anti-RNP 70	4/32 (12,5%)	N.A.
Anti-SSA	15/32 (47%)	N.A.
Anti-SSB	6/32 (19%)	N.A.
Anti-Smith	5/32 (16%)	N.A.
Anticardiolipine auto-antibodies IgG	3/32 (10%)	N.A.
Anticardiolipine auto-antibodies IgM	5/32 (16%)	N.A.
Lupus anticoagulant	32/32 (100%)	N.A.
Anti-b2 glycoprotein IgG	3/29 (10%)	N.A.
Anti-b2 glycoprotein IgM	3/29 (10%)	N.A.

SLEDAI-2 K = SLE Disease Activity Index 2000; SDI = Systemic Lupus International Collaborating Clinics/American College of Rheumatology Damage Index; NPSLE = neuropsychiatric SLE.

### 2.2 Imaging protocol and QSM reconstruction

All subjects were scanned on a 3 T MRI scanner (Achieva; Philips Healthcare). The scan protocol consisted of a short survey scan followed by 3D T_1_-weighted images (voxel size = 0.88 × 0.88 × 1.20 mm, TR/TE = 9.8/4.6 ms) and single-echo T_2_*-weighted images (voxel size = 0.78 × 0.78 × 0.78 mm, TR/TE = 45/31 ms, flip angle 13°). The QSM maps were reconstructed from the magnitude and phase data using STI Suite (Li et al., 2014). A Laplacian-based method was used for phase unwrapping, followed by background field removal using VSHARP (Li et al., 2011, Schweser et al., 2011, Wu et al., 2012). Finally, dipole inversion was performed using iLSQR (Li et al., 2011).

### 2.3 Registration and regional QSM analysis

**Fig. 1:**
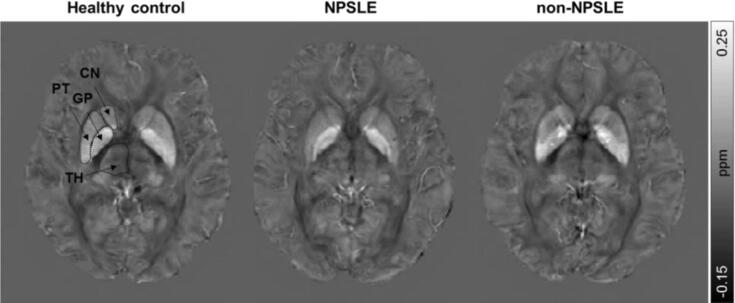
Representative axial QSM images of a healthy control, NPSLE patient and non-NPSLE patient. The regions of interest are indicated in the healthy control. TH = Thalamus; CN = Caudate nucleus; PT = Putamen; GP = Globus pallidus.

3D T_1_-weighted images were registered to the MNI152 template using registration tools contained in FSL (FMRIB's Software Library v6.0, www.fmrib.ox.ac.uk/fsl): FSL’s affine and non-linear registration algorithms, FLIRT and FNIRT, respectively (Jenkinson et al., 2002, Woolrich et al., 2009). Tissue masks of the thalamus, caudate nucleus, putamen, and globus pallidus were segmented based on the 3D T_1_-weighted images using FIRST (FMRIB’s integrated registration and segmentation tool) and subsequently transformed to the MNI152 template space using the conversion matrix acquired from these registrations. Tissue volumes from the thalamus, caudate nucleus, putamen and globus pallidus were calculated in subject space as well as MNI152 space. QSM maps were registered to 3D T_1_-weighted images using a rigid body transformation (elastix (Klein et al., 2010), v4.800, http://elastix.isi.uu.nl). The resulting images were subsequently transformed to the MNI152 template space using the conversion matrix calculated from the 3D T_1_ registration. Mean susceptibility values of the thalamus, caudate nucleus, putamen, and globus pallidus were calculated for each subject from the registered QSM maps in MNI space (Fig. 1). In addition, to rule out more focal differences in QSM maps between SLE patients and controls we used voxel based QSM analysis on the thalamus, caudate nucleus, putamen, and globus pallidus (as defined by the subcortical Harvard-Oxford atlas (Frazier et al., 2005). The pipeline was an adapted form of the FSL-VBM pipeline (Douaud et al., 2007, Good et al., 2001). QSM maps were transformed to MNI152 standard space using non-linear registration (Andersson and Smith, 2007). The resulting images of 20 control subjects and 20 patients (randomly selected) were averaged and flipped along the x-axis to create a left–right symmetric study-specific QSM template. All native QSM maps from the 20 controls and 44 patients included in this study were registered to this study-specific template and modulated with the Jacobian determinant to correct for local expansion or contraction due to the non-linear component of the transformation. The latter is a common step in the standard FSL-VBM pipeline (Douaud et al., 2007, Good et al., 2001) and was done because we were primarily testing for iron content in subcortical structures as opposed to the susceptibility of each transformed voxel. The modulated QSM maps were smoothed with an isotropic Gaussian kernel with a sigma of 3 mm. Finally, a voxel-wise GLM was applied using permutation-based non-parametric testing, correcting for multiple comparisons across space (Winkler et al., 2014).

The reported susceptibility values were inherently referenced to the mean susceptibility value of the whole brain. This was due, in part, to the fact that the frequency shift was referenced to the whole brain mean frequency and the phase filtering process employed using the spherical mean value filters (Li et al., 2014).

### 2.4 Histology of post-mortem brain tissue

Formalin-fixed paraffin-embedded tissue blocks that included the putamen and globus pallidus were collected from three additional SLE patients without known neuropsychiatric complaints (two females and one male; 25/23/20 years old). Also, clinical data as SLEDAI-2k and SDI was not known. The tissue blocks were serially cut into 5 µm sections and deparaffinized and dehydrated in graded series of xylene and ethanol. One section per subject was used for histochemical detection of iron following a previously described protocol (van Duijn et al., 2013). In short, sections were incubated for 30 min in 1% potassium ferrocyanide in a 25 °C water bath, washed followed by 60 min incubation in methanol with 0.01 M NaN_3_ and 0.3% H_2_O_2_ in a 25° water bath. Subsequently, sections were washed with 0.1 M phosphate buffer followed by 30 min incubation in a solution containing 0.025% 3′3-diaminobenzidine-tetrahydrochloride (DAB, (DakoCytomation)) and 0.005% H_2_O_2_ in 0.1 M phosphate buffer in a 25° water bath. The reaction was stopped by washing.

Two consecutive sections adjacent to the section stained for iron were stained for microglia (anti-Iba1 polyclonal rabbit; 1:1000; Wako Chemicals USA) and astrocytes (anti-human GFAP monoclonal mouse (6F2); 1:1000; DakoCytomation, Glostrup, Denmark). All sections were treated with 0.3% H_2_O_2_ in methanol to block endogenous peroxidase activity. This step was followed by an antigen retrieval step that depended on the primary antibody. For microglia, sections were boiled in EDTA, pH 8.5, for 15 min in a microwave and cooled down for 1 h. Sections stained for astrocytes were boiled in citrate buffer, pH 6, for 15 min and cooled down for 1 h. Primary antibodies were incubated overnight at room temperature. The secondary antibody (for microglia: swine anti rabbit biotin (1:400; DAKO) and for astrocytes: rabbit anti mouse biotin (1:200; DAKO))) was incubated for one hour followed by a 30 min incubation with avidin–biotin complex (ABC, Vector Labs, CA, USA). Signal enhancement was completed by immersion in DAB. The sections were counterstained with Harris Haematoxylin, dehydrated, cleared and mounted with Entallan. The slides were digitized using an automatic bright field microscope (Philips Ultra Fast Scanner, Philips, Netherlands) for microscopic evaluation.

### 2.5 Statistical analyses

Patients with SLE and age-matched controls were compared with respect to age, sex and susceptibility values using chi-square tests for categorical variables, and independent *t*-test and ANOVA as the continues variables were normally distributed. Correlations between clinical variables (SLEDAI, SDI and the presence of autoantibodies) and susceptibility values were assessed with Pearson’s correlation tests. All statistical analyses were performed with SPSS version 25.0 for Windows (IBM Corp. Released 2017. IBM SPSS Statistics for Windows, Version 25.0. Armonk, NY: IBM Corp.). Bar graphs and scattered plots were generated using GraphPad Prism 8 for windows, GraphPad Software, USA.

## 3 Results

**Fig. 2:**
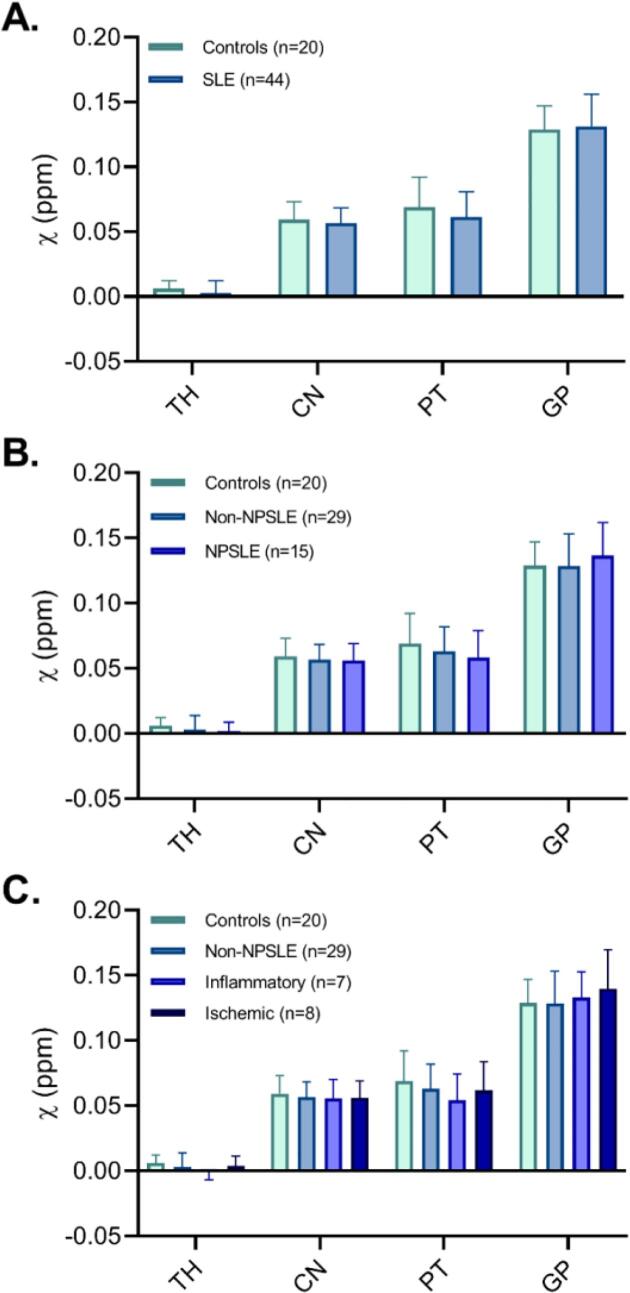
Quantitative magnetic susceptibility (χ in ppm inherently referenced to whole brain) of age-matched controls and SLE patients. No significant differences were found between SLE patients and controls, nor between the different subgroups of SLE based on neuropsychiatric status (non-NPSLE and NPSLE) or clinical phenotype (inflammatory and ischemic NPSLE) and controls. Actual values are given in Supplementary Table 3. TH = Thalamus; CN = Caudate nucleus; PT = Putamen; GP = Globus pallidus.

Representative QSM images of a healthy control, NPSLE and non-NPSLE patient are displayed in Fig. 1. Mean susceptibility values of the thalamus, caudate nucleus, putamen and globus pallidus for the SLE and healthy control group are displayed in Fig. 2. No significant differences were found when comparing susceptibility values between SLE patients and healthy controls in any of the regions of interest. To further investigate whether subcortical iron levels are associated with the attribution of the NP complaints to SLE, the SLE group was stratified into NPSLE and non-NPSLE subgroups, but we did not observe any significant differences in any region of interest. Stratification of only the NPSLE patients into inflammatory and ischemic phenotypes was done to investigate the potential link between neuroinflammation and subcortical iron levels. Again, no significant differences in susceptibility values were found between these two groups. Although all analyses were done in MNI152 space, we performed a volumetric comparison of the individual regions of interest to exclude confounding effects resulting from volumetric differences. Thalamic volumes significantly larger in SLE patients compared to controls (p = 0.012 for volumes assessed in subject space, p = 0.014 for volumes in MNI space). However, comparison of susceptibility values of the thalamus corrected for volume did not affect the results (data not shown). Lastly, to rule out more focal differences in QSM maps between SLE patients and controls we performed a voxel based QSM analysis on the thalamus, caudate nucleus, putamen, and globus pallidus. However, also voxel-based comparisons did not show any significant differences between SLE patients and controls (data not shown).

**Fig. 3:**
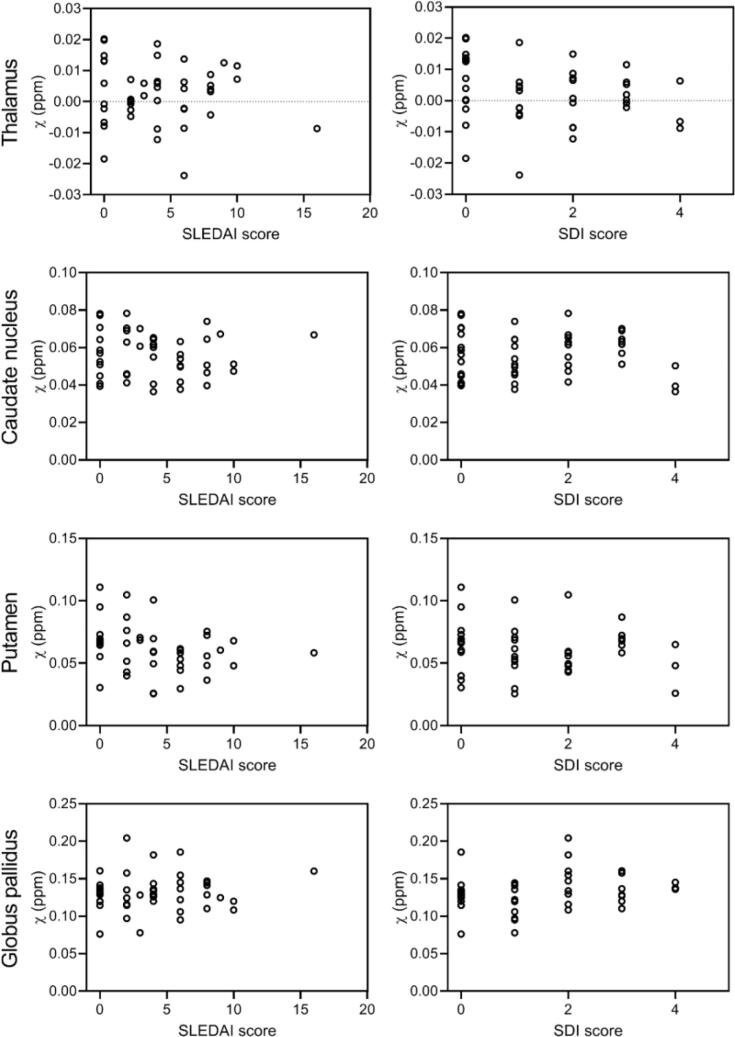
Correlation plots of susceptibility values, clinical scores for disease activity (SLEDAI-2 K) and damage due to SLE (SDI). When all patients with SLE were pooled together, no significant correlations were found in any region of interest. Similar results were found when stratifying patients according their neuropsychiatric status (non-NPSLE and NPSLE) or clinical phenotype (inflammatory and ischemic NPSLE).

To investigate the link between subcortical iron accumulation and disease activity, correlation analysis was performed between susceptibility values and SLEDAI-2K scores. When all patients with SLE were pooled together, no significant correlations were found in any region of interest (Fig. 3, left column). Similar results were found when stratifying patients according their neuropsychiatric status (non-NPSLE and NPSLE) or clinical phenotype (inflammatory and ischemic NPSLE). Also the link between subcortical iron accumulation and damage due to SLE, represented as SDI score, was investigated, but no significant correlations were found in any of the regions of interest (Fig. 3, right column). Stratifying patients as done above resulted in similar results. Finally, the presence of auto-antibodies also did not correlate with susceptibility values.

**Fig. 4:**
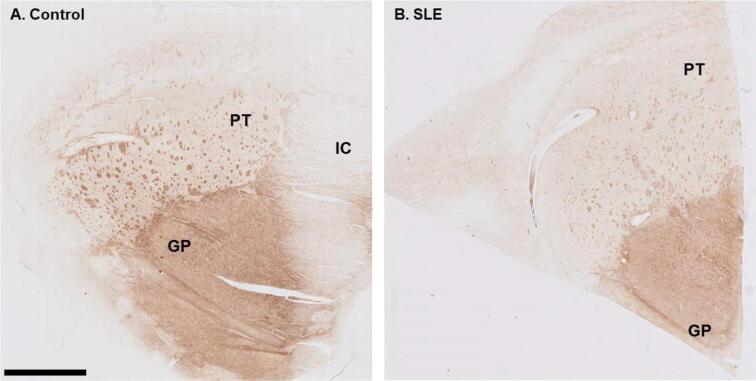
Macroscopic overview of iron stained putamen and globus pallidus of a control and SLE brain. The globus pallidus showed higher staining intensity compared to the putamen. Within the putamen small areas of increased iron staining were found originating from myelinated fiber bundles traversing the putamen. GP = Globus pallidus; PT = Putamen; IC = Internal capsule. Scale bar size = 5 mm.

To further explain the in vivo observations, post-mortem histological analyses of brain tissue from SLE patients were performed. Areas that were investigated included the putamen and globus pallidus. Macroscopic examination of the iron staining showed higher staining intensity in the globus pallidus compared to the putamen, closely resembling the in vivo susceptibility findings (Fig. 4). Within the putamen small well-defined areas of increased iron staining were found, originating from myelinated fiber bundles traversing the putamen. The macroscopic iron distribution in a control subject showed a similar pattern.

**Fig. 5:**
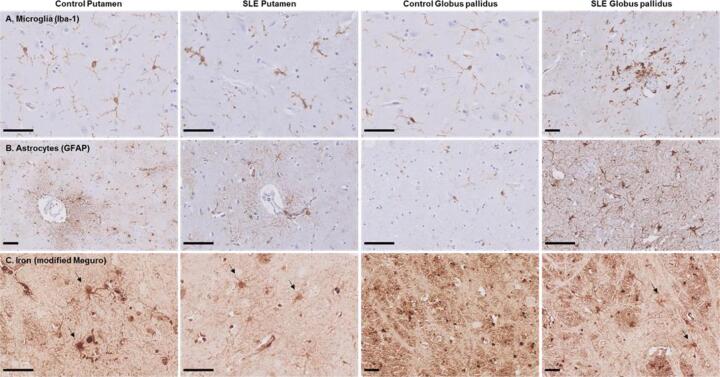
Microglia, astrocytes and iron in the putamen and globus pallidus of SLE patients and normal control. (A) In control putamen and globus pallidus microglia with thin ramified processes were found. SLE patients showed dystrophic microglia characterized by beaded, twisted and fragmented processes. Within the globus pallidus of one SLE patient a small area with activated microglia surrounding a blood vessel was found. (B) Astrocytes, on the other hand, showed in both control and SLE brain a normal morphology with a dense network of finely branching processes especially close to vessels. In the control globus pallidus only a few astrocytes were found. (C) The modified Meguro staining showed that iron was predominantly found in oligodendrocytes and myelin and, to a lesser extent, in neurons, microglia and astrocytes (arrows) in both control and SLE brain. Scale bar size = 50 µm.

Microscopically, dystrophic microglia were present in all three SLE patients, characterized by beaded, twisted and fragmented processes (Fig. 5A). A small region with activated microglia surrounding a blood vessel was found within the globus pallidus of one SLE patient. However, in these particular cases, no signs of significantly increased inflammation were found. In contrast, microglia in control brain showed a classical homeostatic morphology observed as ramified microglia exhibiting highly branched processes. Astrocytes, on the other hand, showed in both SLE and control brain a normal morphology with a dense network of finely branching processes especially close to vessels (Fig. 5B). The iron staining showed that iron was predominantly found in oligodendrocytes and myelin and, to a lesser extent, in neurons, microglia and astrocytes (Fig. 5C, arrows). The amount of iron in cells morphologically resembling microglia and astrocytes was not visibly different in SLE brains compared to control brains.

## 4 Discussion

Our main aim in this study was to explore the potential link between iron accumulation and neuroinflammation in SLE, following several findings that point to such a link in diseases as Alzheimer’s disease (Ayton et al., 2017, van Bergen et al., 2016a), Parkinson’s disease (Acosta-Cabronero et al., 2017, Langkammer et al., 2016), and Huntington’s disease (Kaunzner et al., 2019, Langkammer et al., 2013, Macerollo et al., 2014, van Bergen et al., 2016a). Our tool of choice for this exploration was QSM, based on the assumption that increases in local tissue susceptibility reflect primarily increases in local tissue iron concentration (Deistung et al., 2013, Langkammer et al., 2012, Stüber et al., 2014). We hypothesized that similarly to the diseases previously mentioned, SLE patients and in particular NPSLE patients would have increased numbers of activated microglia co-localizing with iron, which in turn would be reflected in increased local susceptibility.

Increased susceptibility values in the basal ganglia of patients with for example multiple sclerosis and Huntington’s disease were reported in several studies (Haider et al., 2014, Langkammer et al., 2013, Macerollo et al., 2014, van Bergen et al., 2016b), whereas currently only one study used QSM to explore brain abnormalities in SLE and NPSLE patients (Ogasawara et al., 2016). This study reported increased susceptibility values in the putamen of NPSLE patients compared to non-NPSLE patients and increased susceptibility values in the globus pallidus of both NPSLE and non-NPSLE patients compared to controls. Susceptibility values of the putamen were also correlated with disease duration in NPSLE patients (Ogasawara et al., 2016). However, in our study susceptibility values of SLE patients and age-matched controls showed that iron levels in the thalamus and basal ganglia are not changed due to the disease. Furthermore, none of the SLE subgroups analyzed showed higher susceptibility values, including the inflammatory NPSLE group which is the one associated most with neuroinflammation. However, a lack of significance does not necessarily prove that susceptibility values are not changed in SLE patients. Therefore, we looked at the confidence intervals of our results, to examine the magnitude of any potential effect. In our study, the mean difference of the putamen between NPSLE patients and controls was −0.011 ppm (CI −0.028, 0.007). Notably, although the mean difference of 0.011 ppm found between the putamen of NPSLE patients and controls found by Ogasawara (Ogasawara et al., 2016) falls outside the bounds of our confidence interval, we cannot rule out that NPSLE patients may have relevantly different susceptibility values compared to controls of up to 0.007 ppm. However, the differences in susceptibility values between NPSLE patients and controls we found is small compared to QSM studies in Alzheimer’s disease (AD) (Ayton et al., 2017, van Bergen et al., 2016a) and Huntington’s disease (HD) (van Bergen et al., 2016b, Chen et al., 2019, Domínguez D et al., 2016). As the reported mean susceptibility differences of the putamen between HD patients and controls ranges from 0.032 ppm in one study (Domínguez D et al., 2016) to 0.023 ppm in the other (Chen et al., 2019), it seems that the effect is probably smaller in SLE compared to neurodegenerative disease such as HD in which iron clearly plays a role.

Comparison of the methodology and patient cohort of the previous and present study did reveal some important differences. First, several methods exist to reconstruct QSM data and, in general, it has been observed that the susceptibility values vary depending on the employed inversion method as well as the selection of a reference region (Langkammer et al., 2018). The susceptibility values reported by Ogasawara et al are reconstructed using the MEDI toolbox (Ogasawara et al., 2016, Liu et al., 2012) whereas our results are derived from QSM maps reconstructed using the STI Suite toolbox (Li et al., 2014). Similar to our results, highest values are reported for the globus pallidus corresponding to the literature. In contrast to our results, which concur with the literature (van Bergen et al., 2016b, Lim et al., 2013, Acosta-Cabronero et al., 2017, Langkammer et al., 2016, Langkammer et al., 2013), in both control subjects and patients the susceptibility values obtained from the other basal ganglia structures are systematically higher than those reported in the literature. Susceptibility values of the thalamus are consistently reported to be lowest compared to the other basal ganglia structures with values ranging from −0.02 to 0.016 ppm (Jeltsch-David and Muller, 2014, van Bergen et al., 2016b, Hochberg, 1997, Liu et al., 2012, Lim et al., 2013). Remarkably, Ogasawara et al reports about a 10-fold higher susceptibility value for the thalamus in both control subjects and patients. As mentioned, currently a standard pipeline for QSM reconstruction is lacking, complicating the comparison of susceptibility values across studies. In addition, using a reference value for QSM is preferred, however, this information is missing in the work of Ogasawara et al. that could further explain the differences in reported susceptibility values. In the work of Ogasawara et al., the globus pallidus was manually segmented into a lateral and medial region, and significant susceptibility differences between groups were found in the lateral part. Visual inspection of the globus pallidus in our cohort showed that the contrast within the globus pallidus is highly heterogeneous and in many cases patchy, both in controls as well as in patients, making a clear delineation of the two parts of the globus pallidus all but impossible. This patchy contrast is likely explained by combination of factors typically found in this age group: enlarged perivascular spaces, calcification and iron accumulation, that are non disease-specific.

Secondly, as shown by Ercan et al. (2016), cell-specific microstructural alterations as measured with diffusion-weighted magnetic resonance spectroscopy (DW-MRS) are correlated with disease state and activity. More specifically, the diffusivity of the glial metabolites creatine and choline were significantly higher in SLE-active patients and are hypothesized to reflect inflammation-mediated morphological changes of microglia and astrocytes (Ercan et al., 2016). From studies in other neurodegenerative diseases it is known that inflammation is correlated with disease severity and might be more pronounced in the progressive or active stage of the disease (Kaunzner et al., 2019, Crotti and Glass, 2015, Heneka et al., 2015). Similar results are reported regarding iron accumulation, where iron accumulation is positively correlated with disease severity in patients with Alzheimer’s and Huntington’s disease (Ayton et al., 2017, van Bergen et al., 2016a, van Bergen et al., 2016b). However, we did not observe any differences in susceptibility values between SLE patients and control subjects, nor did we find any correlation with disease activity score. It is possible, that within the range of disease activity scores of patients in this particular cohort, glial activation was moderate compared to the study from Ogasawara et al. (2016) where the overall SLEDAI was higher, explaining why we did not observe any differences and or correlations.

Histological examination of post-mortem brain tissue including the putamen and globus pallidus of SLE patients provides a potential link with the in vivo findings as the iron staining did not show significant increased staining intensity compared to control brain tissue. The amount of iron in cells morphologically resembling microglia and astrocytes was not visibly changed in SLE brains compared to control brains. From a global inflammatory brain disease such as multiple sclerosis, we know that increased iron accumulation is found in microglia in conjunction with axonal and neuronal degeneration (Haider et al., 2014). In our study, we found dystrophic microglia in SLE post-mortem brain tissue, however the number of cells was not obviously increased as compared to control brain tissue and no signs of reactive astrocytes and neurodegeneration were present. We hypothesize that, especially in patients with relatively low disease activity, iron accumulation in solely microglia and maybe astrocytes is not sufficient to affect tissue susceptibility as the amount of glia cells is not significantly increased. Additionally, QSM might not be sensitive enough to detect iron accumulation in solely microglia without additional axonal and neuronal damage as seen in other neurodegenerative diseases in which neurodegeneration, neuroinflammation and iron accumulation are clearly present (Bulk et al., 2018, Simmons et al., 2007).

Previous imaging studies in NPSLE and SLE predominantly reported differences in white matter between patients and controls, such as decreased magnetization transfer ratio, increase in mean diffusivity and decrease in fractional anisotropy of DTI (Ercan et al., 2015, Ercan et al., 2016, Magro-Checa et al., 2016). These diffuse findings (as opposed to “visible” findings such as atrophy and white matter hyperintensities) point towards neuroinflammation as a mechanism that increases the amount of free water in tissue as a result of intracellular edema (Sibbitt et al., 2010, Brooks et al., 2010). Some studies showed basal ganglia involvement in NPSLE, as for example decreased N-acetylasparate, suggesting decreased neuronal function within the putamen and caudate nucleus (Lim et al., 2000). However, as the vast majority of studies in SLE and NPSLE focused on the investigation of white matter involvement (Lee et al., 2012, Zhang et al., 2005, Ercan et al., 2015, Ercan et al., 2016, Magro-Checa et al., 2016), it would be of interest to investigate iron accumulation in similar regions and correlate with other tissue and physiological deficits found in white matter. Obtaining unbiased susceptibility values from QSM in white matter is challenging due to the orientation dependency of QSM, and both white matter tract orientations as well as head orientation with respect to the magnetic field should be taken into account. This can potentially be eliminated by including the fiber orientation information from diffusion tensor imaging (Li et al., 2014). In addition, comparing mean susceptibility values of basal ganglia substructures between SLE patients and controls gives information on diffuse brain changes whereas in white matter, local changes such as lesions are also present. Previous MRI studies have shown that white matter hyperintensity lesions are present in 70% of the SLE patients. Recent studies in multiple sclerosis showed the usefulness of QSM in the detection of chronic active lesions as these lesions show a hyperintense rim on QSM which colocalizes with iron containing microglia (Haider et al., 2014). In SLE such a phenomenon has not been encountered, but the presence of iron in lesions or lack thereof could provide information on the presence of persistent inflammation activity in for example relapsing patients.

A challenging aspect of NPSLE research is the heterogeneity and the small sample size due to the low prevalence of the disease. Our study included 44 SLE patients and 20 age-matched controls, making it of comparable size to previous studies reporting positive results (Ogasawara et al., 2016). Stratifying patients into subgroups, and especially stratifying the patients into primary ischemic and primary inflammatory decreased the number of cases per groups significantly, limiting the possibility of reaching statistical power. Given the complexity and low prevalence of specific phenotypes, collaborative efforts such as multicenter studies and meta-analyses of existing data are desirable approaches. NPSLE poses significant challenges for both approaches. Diagnosis for NPSLE is primarily based on the ACR 1999 criteria, but the threshold for attribution of each criterion to NPSLE varies greatly across clinics. In such a heterogeneous, rare and difficult to diagnose disease as NPSLE, the most desirable way to increase the study cohorts is to carefully plan a multicenter prospective study with as aligned a diagnostic process as possible across centers. We hope that with such well-planned efforts, a reliable pooling of datasets in NPSLE will be made possible, with a potential significant increase in the explanatory power of MRI for the underlying mechanisms of NPSLE. The same applies for post-mortem studies on SLE and NPSLE, as comparison of in vivo findings with post-mortem evidence is ideally done within the same subjects. Unfortunately, due to the low prevalence and rarity of brain donations from SLE patients and specifically NPSLE patients, our study used brain tissue from different patients than those included in the in vivo study and SLEDAI-2K and SDI scores were not known. Nevertheless, the results provide important information for the possible interpretation of the in vivo findings.

In conclusion, this study did not find susceptibility changes in the thalamus and basal ganglia of SLE patients on MRI. In that respect NPSLE might behave differently than other neuroinflammatory and neurodegenerative diseases such as multiple sclerosis. However, we do not exclude the possibility of iron induced magnetic susceptibility changes in NPSLE. As conclusive evidence is lacking, this should be further investigated and may offer opportunities for differential diagnosis and further insights into the diverging pathomechanisms of neuroinflammatory diseases. More data from both post-mortem (NP)SLE brains as well as more in vivo data from independent sources such as iron concentrations in cerebrospinal fluid are needed to obtain a better picture of iron involvement in (NP)SLE.

## CRediT authorship contribution statement

**Marjolein Bulk:** Conceptualization, Data curation, Formal analysis, Investigation, Validation, Visualization, Writing - original draft. **Thijs van Harten:** Software, Methodology. **Boyd Kenkhuis:** Investigation, Validation. **Francesca Inglese:** Resources, Data curation. **Ingrid Hegeman:** Investigation, Validation. **Sjoerd van Duinen:** Investigation, Validation. **Ece Ercan:** Resources, Methodology. **César Magro-Checa:** Investigation, Writing - review & editing. **Jelle Goeman:** Methodology. **Christian Mawrin:** Resources. **Mark van Buchem:** Writing - review & editing. **Gerda Steup-Beekman:** Investigation, Writing - review & editing. **Tom Huizinga:** Writing - review & editing. **Louise van der Weerd:** Conceptualization, Supervision, Writing - original draft. **Itamar Ronen:** Conceptualization, Funding acquisition, Supervision, Writing - original draft.

## Declaration of Competing Interest

The authors declare that they have no known competing financial interests or personal relationships that could have appeared to influence the work reported in this paper.
