# Supplementary material for: Quantitative susceptibility mapping in the thalamus and basal ganglia of systemic lupus erythematosus patients with neuropsychiatric complaints
Source: Neuroimage Clin. 2021 Mar 22;30:102637. doi: 10.1016/j.nicl.2021.102637 (PMC8053812; doi:10.1016/j.nicl.2021.102637)
Supplement: Supplementary data 1 [file mmc1.docx]

### **Supplementary material**

**Supplementary Table 1. Neuropsychiatric symptoms in NPSLE patients and non-NPSLE patients.**

| ***NPSLE patients*** *(n = 15)* | | ***Non-NPSLE patients*** *(n = 29)* | |
| --- | --- | --- | --- |
| *1999 ACR NPSLE syndromes^a^ (n, % total)* | | *Diagnoses (n, % total)* | |
| Headache | 1 (5) | Headache | 12 (24) |
| Seizure disorder | 1 (5) | Neurological diagnoses^c^ | 6 (12) |
| Cerebrovascular disease | 8 (38) | Cognitive dysfunction | 6 (12) |
| Myelopathy | 1 (5) | Mood disorder/ other psychiatric disorders | 12 (24) |
| Cognitive dysfunction | 2 (10) | Not objectified complaints^d^ | 7 (14) |
| Mood disorder | 2 (10) | Other | 7 (14) |
| Acute confusional state | 1 (5) |  |  |
| Polyneuropathy | 2 (10) |  |  |
| Cranial neuropathy | 1 (5) |  |  |
| Other*^b^* | 2 (10) |  |  |

^a^ Patients with neuropsychiatric symptoms attributed to SLE. All neuropsychiatric symptoms are defined, but not restricted, according to those alternative diagnoses and the NPSLE definitions included in the 1999 ACR nomenclature; ^b^ Other NPSLE syndromes were: pyramidal tract syndrome (n = 1), lethargia (n = 1); ^c^ Neurological diagnoses, including e.g. polyneuropathy and epilepsy not due to SLE; ^d^ Not objectified complaints mainly included cognitive complaints.

**Supplementary Table 2. Use of medication at time of MRI assessment in all patients.**

|  | **All patients**  n = 44 |
| --- | --- |
| ***Medication at time of MRI (n, %)*** |  |
| *Immunosuppressive therapy* |  |
| Corticosteroids | 24 (55) |
| NSAID | 7 (16) |
| Hydroxychloroquine | 26 (59) |
| Azathioprine | 5 (11) |
| Methotrexate | 4 (9) |
| Mycophenolate mofetil | 5 (11) |
| Cyclophosphamide | 1 (3) |
|  |  |
| *Secondary prevention* |  |
| Antiplatelet drugs | 11 (25) |
| Vitamin K antagonist | 5 (11) |
| Low molecular weight heparin | 1 (2) |
| Statins | 6 (14) |

**Supplementary Table 3. Actual measured susceptibility values in ppm and corresponding standard deviations.**

|  | Thalamus | | Caudate Nucleus | | Putamen | | Globus Pallidus | |
| --- | --- | --- | --- | --- | --- | --- | --- | --- |
|  | Mean | SD | Mean | SD | Mean | SD | Mean | SD |
| Controls (n=20) | 0.006 | 0.006 | 0.059 | 0.014 | 0.069 | 0.023 | 0.129 | 0.018 |
| SLE (n=44) | 0.003 | 0.001 | 0.056 | 0.012 | 0.061 | 0.019 | 0.131 | 0.025 |
|  |  |  |  |  |  |  |  |  |
| Controls (n=20) | 0.006 | 0.006 | 0.059 | 0.014 | 0.069 | 0.023 | 0.129 | 0.018 |
| Non-NPSLE (n=29) | 0.003 | 0.011 | 0.056 | 0.012 | 0.063 | 0.019 | 0.129 | 0.025 |
| NPSLE (n=15) | 0.002 | 0.007 | 0.056 | 0.013 | 0.058 | 0.021 | 0.137 | 0.025 |
|  |  |  |  |  |  |  |  |  |
| Controls (n=20) | 0.006 | 0.006 | 0.059 | 0.014 | 0.069 | 0.023 | 0.129 | 0.018 |
| Non-NPSLE (n=29) | 0.003 | 0.011 | 0.056 | 0.012 | 0.063 | 0.019 | 0.129 | 0.025 |
| Inflammatory (n=7) | -0.001 | 0.006 | 0.056 | 0.014 | 0.054 | 0.020 | 0.133 | 0.020 |
| Ischemic (n=8) | 0.004 | 0.007 | 0.056 | 0.013 | 0.062 | 0.022 | 0.140 | 0.030 |
